# The Food and the Teeth

**Published:** 1865-11

**Authors:** 


### THE FOOD AND THE TEETH.

**Observations on the Inorganic Constituents of the Food of Children, as connected with the Decay of the Teeth, and the Physical Constitution of Women in America.**

[The following very valuable and instructive paper was read before the District Medical Society for the County of Mercer, N. J., some years ago, by the late JAMES PAUL, M. D.. of Trenton. There is so much in it worthy the consideration of the profession and the public, that we deem it of sufficient importance to reproduce it.—ED. MED. AND SURG. REP.]

The subject to which I have the pleasure of directing your attention is, not only in a physiological point of view, one of interest, but in its application to the preservation of health—the tendency to improve the general condition and physical constitution of the human family inhabiting this great continent—a continent abounding, as it does, in all the productions which a bountiful Creator, in his beneficence, bestows on man—can not be otherwise than of great and paramount importance.

At a period somewhat now remote, the celebrated naturalist, BUFFON, alluding to the animals of this continent, advanced the following opinions:

1st. That the animals common both to the Old and New Worlds are smaller in the latter.

2d. That those belonging to the New are on a smaller scale.

3d. That those which have been domesticated in both, have degenerated in America.

4th. That, on the whole, it exhibits fewer species.

These opinions, Mr. JEFFERSON, in his "Notes on Virginia," undertook, and it is generally considered suceessfully, to controvert; yet, however repugnant to the general idea the opinion as to the tendency of those animals which have been domesticated in America from other countries to degenerate, it is an undeniable and much to be regretted fact, that the human family, and more particularly the female portion of that family, have declined in the vigor and strength of their physical constitution.

I wish not to be misunderstood: I say it is a melancholy fact, too well known to the observant physiologist, that increase of strength, and development of frame, have not been attained by the intermarrying of members of the human family of different nations on this continent; but the reverse is too observable: the physical frame of the female sex has degenerated—calling loudly for the aid of science to arrest an evil of so much magnitude.

Let us for a moment contemplate the female form, as seen on this broad continent. In no country in the world are children more fair and beautiful; and as the young girl grows up to womanhood, we see in her a full realization of that being forming in the hands of Divinity, portrayed by the poet, as seen by Adam in his dream:

> "Under his forming hands, a creature grew,
> Manlike, but different sex; so lovely fair,
> That what seemed fair in all the world, seemed now
> Mean, or in her summed up, in her contained,
> And in her looks;"—

We see this young and lovely being—the forehead well developed—the countenance, rather elongated, relieved of the harsher outline of some of the European nations—with fragile form, and small, yet well-developed bust, flitting for a few short years among us, and then—yes, then there comes a change. Ere five and twenty summers pass, this flower begins to fade—the rounded form shrinks—the bloom of health decays; and if she escape the fell destroying angel's death-like grasp, a wreck of former self remains.

Why should this be so? The robust of other countries come to this continent—they live in comfort—their food is excellent in quality—their progeny is like themselves—but even now, in the very first generation, does the degenerating process make itself manifest—the teeth begin to decay; and girls, while yet children, have to visit the dentist to have them cleansed, scraped, and plugged.

Now this brings us at once to the head and front of our subject; and if we can point out the first cause of this decay of what should be as strong as adamant, it may be the means of helping us in our investigation. That there is something radically wrong in our system of rearing the young, to which this misfortune is in a great measure owing, I am free to confess, is my firm opinion. I would indeed it were in my power, in pointing out the evil, to be as successful in detailing the cause, that we may apply the remedy. Still, although perhaps unable to accomplish all I wish, my observations may not be without their weight, and induce others, more observant, more scientific, and more competent to the task, to follow up an investigation so fraught with advantage to our fellow-beings.

It is certainly to be deplored that the females of this continent, descendants of European parents, should be so much afflicted with caries of the teeth—the decay of parts formed of substances which enter into the composition of some of our hardest minerals—marble, bone earth and fluorspar; and this decay unfortunately occurs in early life—in girls yet at school; and many a young woman, ere she has attained a marriageable age, has had to replace the natural with the unnatural, though more enduring enamel of the artist's formation. This ought not to be: God made all mankind alike; in no portion of the earth are nations found who lose their hands, or feet, or tongue, or eyes; and there can be no cause why the inhabitants of this land should lose their teeth. It is not so in the olden countries from whence the progenitors of the present race have come; nor is it so in the West India Islands, which may almost be considered as part of this great continent. So excellent is the structure of the teeth of savage nations, that some tribes in Africa, I think the Mocoes and Mundingoes, file all the front teeth, so that they shall be separated and form sharp points, the better to tear the uncooked animal food.

One cause of this affliction is, in the mind of many, attributed to the great and sudden changes of temperature experienced on this continent—the thermometer rising and falling 20, 30, and even 40 degrees in twelve hours. But if attributable to these sudden changes, we know that sudden expansion by means of heat, or sudden contraction by means of cold, causes the particles of which bodies are composed to tear themselves asunder; consequently to crack, break and fall in pieces. But this is not the case with the teeth of our females ; a caries or decay commences most generally in the side of the tooth, extending to the enamel, which is sometimes involved in the destruction; at other times, it is left a crust or shell to snap and break off in small pieces, when unable to resist the pressure of whatever may be placed against it; besides, the teeth are for the most part sheltered from these sudden changes, and kept at a temperature nearly amounting to blood-heat at all seasons. I do not think we can place the general destruction of the teeth, and consequent affliction of the females of America, to this cause. I fear we must rather look for it to constitutional weakness, and this constitutional weakness to a deficiency of the inorganic or earthy constituents being taken into the system, more particularly at an early period of life.

If I am correct in this opinion, and reason, philosophy, and a thorough examination of physiological facts in both the animal and vegetable economy, tend far to bear out these views, then if we would try and correct this lamentable state of things, let us commence at the very beginning, and make ourselves acquainted by examining the structure and composition of the teeth, and then we shall be more able to understand what is required to aid nature in their formation and consequent preservation.

First, then, let us make ourselves acquainted with the structure and composition of the teeth. The teeth are nearly allied to bone in structure; both having earthy deposits, intermixed with fibres and cells of gelatine, which, by consolidation, gives form and strength—in the case of bone, to bear the weight of the various parts, and afford protection to the different organs of the body ; and in the case of teeth, to cut and grind the food required for the formation, support, and reparation of its various parts.

Now, teeth are composed of three different substances, and these three are disposed according to the purposes required of them; they are, *cementum* or *crusta petrosa*, *dentine* (known as ivory in the tusk of the elephant), and *enamel*. The *ce-*

*mentum* or *crusta petrosa*, corresponds in all especial particulars with bone; possessing its characteristic *lacunæ* or small cavities, and being traversed by vacsular medullary canals, whenever it occurs of sufficient thickness; it is the first covering of the young teeth, and may be said to invest the fang of the tooth which enters the alveolar process of the jaw. The *dentine*, or ivory, consists of a firmer substance, in which inorganic or mineral matter predominates, though to a less degree than in *enamel*. It is traversed by a vast number of very fine cylindrical, branching, wavy tubuli, which commence at the pulpy cavity, and radiate toward the surface. The diameter of these tubuli, at their largest part, averages about one 10,000th of an inch: their smallest are immeasurably fine; so much so, that they cannot possibly receive blood, but it is surmised that like the canaliculi of bone, they imbibe fluid from the vascular lining of the pulp-cavity, which aids in the nutrition of the tooth. The *enamel* is composed of solid prisms of fibres, about the one 5,600th of an inch in diameter, arranged side by side, and closely adherent to each other: their length corresponds with the thickness of the layer which they form; and the two surfaces of this layer present the ends of the prism, which are usually more or less hexagonal. In the perfect state, the enamel contains but an extremely minute quantity of animal matter. In the centre of the tooth is the soft pulpy cavity, which affords a bed for the blood-vessels and nerves which supply it with life and sensibility.

I shall not enter more minutely into the structure of the teeth, but may briefly state that, like all other structures of the animal body, the component parts are derived and deposited from the blood, by that mysterious and incomprehensible power that selects and deposits the necessary constituents in the formation of the several portions, according to the use required.

Now, in the composition of the teeth, we have first the division into organic and inorganic or earthy matter; and we find that the several substances which enter into the structure of the teeth, differ chiefly as to the earthly matter contained in each.

Chemical analysis of the incisors, or front teeth of man, show that they contain in one hundred parts of each, as follows:

| | Cementum. | Dentine. | Enamel. |
|---|---|---|---|
| Organic Matter | 29.27 | 28.70 | 3.59 |
| Earthly Matter | 70.73 | 71.30 | 96.41 |
| | 100. | 100. | 100. |

These proportions will occasionally differ; in some individuals the organic constituents having less than here stated, amounting in the dentine only to 21. The analysis of bone, however, gives a much larger proportion, viz:

| | |
|---|---|
| Organic Matter | 32.56 |
| Earthly Matter | 67.44 |
| | 100. |

Let us now take a more a complete analysis, showing what earthy constituents enter into to their composition. Analysis of the molar or grinding teeth of man, and of the bones of the arm and leg of a man of forty, show the following proportions:

| Inorganic Matter:— | Dentine. | Enamel. | Bone. |
|---|---|---|---|
| Phosphate of Lime, with traces of Fluate of Lime | 66.72 | 89.82 | 54.61 |
| Carbonate of Lime | 3.36 | 4.37 | 9.41 |
| Phosphate of Magnesia | 1.08 | 1.34 | 1.07 |
| Salts, etc | .83 | .88 | 2.35 |
| Organic Matter | 28.01 | 3.59 | 32.56 |
| | 100. | 100. | 100. |

Thus we see the very great proportion of certain earths that enter into the structure of the teeth and bone of man, the chief substance being the phosphate of lime, familiarly known as bone earth. We find, too, that whereas in ordinary bone the phosphate of lime constitutes only 54 parts in 100, in the enamel of the teeth it is nearly 90 parts in 100 —while the carbonate of lime in bone amounts to 9.41, in the enamel of teeth it is only 4.37; the enamel being literally almost a mineral in substance, having only 3.59 parts of animal matter in 100.

Thus the teeth to be strong and durable, require a large quantity of earthy ingredients, particularly *lime*, to enter into their composition. Let us enquire whence it is derived; and for this we must examine the blood.

To allow of such deposits from the blood, it is first necessary that they should be held in solution in that fluid. You are no doubt aware that the blood circulates to every portion of the body by the action of the heart, which forces a certain quantity, say 2 oz. at every contraction, into the aorta or great canal leading from the left ventricle—that the aorta divides and subdivides into innumerable branches, which are made to ramify to every part of the body, until the extreme branches end in capillary tubes or vessels, the calibre of which is so small as not to allow the red globules or corpuscles of the blood to enter them, but which allows the serous portion to traverse every part of the organized structure, holding in solution all those constituents necessary and requisite for the formation and reparation of its several parts.

In the serous portion of the blood, then, we find contained the constituents required for the composition of bone and teeth—analysis of 1000 parts of healthy human blood giving, according to M. LECANU, the following proportions:

| | | |
|---|---|---|
| Water | 780.15 | 785.58 |
| Fibrine | 2.10 | 3.57 |
| Albumen | 65.09 | 69.41 |
| Coloring matter | 133.00 | 119.63 |
| Crystalizable fat | 2.43 | 4.30 |
| Fluid fat | 1.31 | 2.27 |
| Extractive matter, uncertain | 1.79 | 1.92 |
| Albumen in combination with Soda | 1.26 | 2.01 |
| Chlorides of Sodium and Potassium; Carbonates, Phosphates and Sulphates of Potash and Soda | 8.37 | 7.30 |
| Carbonates of Lime and Magnesia; Phosphates of Lime, Magnesia and Iron; Peroxide of Iron | 2 10 | 1.42 |
| Loss | 2.40 | 2.50 |
| | 1000. | 1000. |

We see by this table, if we subtract or take away the proportion of water amounting to 780 parts, and the coloring matter amounting to 133, we shall leave scarcely 90 parts of organic and earthy material, the salts and earths forming upwards of a 10th—the salts being in proportion to the earths as 4 to 1.

Having then shown the constituent portions of the bones and teeth to be in the blood, the next consideration is, whence are they derived?

Before entering on this subject further, let us for a moment take a broader and more comprehensive view of what must be most interesting to mothers, and of great consequence to the well-being of the infant generation, in a short time, in a very few years, to become in their turn the mothers and fathers of another generation.

The question then presents itself, what is the nourishment or food best adapted and necessary to the wants of an infant, that the foundation may be laid for a strong frame and vigorous constitution? For here, we must recollect, is the starting point in by far the majority of instances. We know that in some cases disease is hereditary—that the offspring unfortunately inherits from the parents constitutional defects; but we also know that more misery, suffering, and constitutional derangement, are entailed on children by want of care, and improper food in the first years of life, by which their hopes of health are blasted, and they are doomed to struggle through a weary life, to be hurried at last into a premature grave.

Now, that the frame—that is, the bones, muscles and other portions of the infant—may be fully developed, it is necessary that it should be supplied with nourishment, containing all the constituents required for this important undertaking. And this nourishment, by the all-wise ordering of Providence, is contained in the milk secreted from the mother's bosom.

The infant is entirely dependent on the nourishment derived from its mother, and nature has wisely ordained that the secretion from the mother is its very best food; for we find in the composition of milk—that is, healthy milk, derived from healthy blood—all those ingredients we have hitherto traced as requisite in the formation of the bones and teeth, and not only in these, but every constituent required for the life and growth of the individual;—milk containing the albuminous, saccharine, oleaginous, saline, and earthy compounds requisite and necessary for the health, strength, and development of the infant child.

How thankful ought we to be to the all-wise and bountiful Giver of all good, for this beneficent, this wonderful provision in nature, by which there shall be secreted from the mother, a fluid so important, having properties blended in intimate connection, to afford the requisite substances for the support, growth and development of her offspring.

An analysis of cow's milk gives the following proportions of the various constituents; that of human milk is not so elaborate, but contains the average of observations taken at fourteen different times from the same individual, by SIMON.

Cow's Milk by M. Haidlen.

| | |
|---|---|
| Water | 873.00 |
| Butter | 30 00 |
| Caseine | 48.20 |
| Milk Sugar | 43.90 |
| Phosphate of Lime | 2.31 |
| Phosphate of Magnesia | .42 |
| Phosphate of Iron | .07 |
| Chloride of Potassium | 1.44 |
| Chloride of Sodium | .24 |
| Soda in connection with Caseine | .42 |
| | 1000. |

Woman's Milk by Simon.

| | |
|---|---|
| Water | 883.6 |
| Butter | 25.3 |
| Caseine | 34.3 |
| Milk Sugar and Extractive Matter | 48.2 |
| Fixed Salts | 2.3 |
| | 1000. |

| | Maximum of 14 observations. | Minimum of 14 observations. |
|---|---|---|
| Butter | 54.0 | 8.0 |
| Caseine | 45.2 | 10 6 |
| Sugar and Extractive Matter | 62.4 | 39.2 |
| Salts | 2 7 | 1.6 |

Now although these amounts will no doubt vary, under every variety of circumstances, according to the *health*, *exercise*, *passions*, and *food* of the mother, yet they show what I particularly wish to impress on your minds, that healthy milk contains all the requisites for the nourishment of the infant—but then it must be *healthy* milk, secreted from healthy blood, and that blood must derive these ingredients from the *food* consumed, otherwise they will be taken up from the structures of the body, and hence the havoc made in nursing females when a due allowance of proper aliment is withheld, and the shrunken body of the famished mother is drained to the last drop, to supply the cravings of the death-like and impoverished offspring.

I have said that the composition of milk in quality and quantity, will vary and depend on circumstances. Now the mental state exerts a surprising influence on this secretion, and much more than is usually supposed. It may not be irrelevant to mention a few of the cases recorded in our journals,* of the influence of strong mental excitement on this secretion.

* From Carpenter's Physiology.

"A carpenter fell into a quarrel with a soldier billeted in his house, and was set upon by the latter with his drawn sword. The wife of the carpenter, at first, trembled from fear and terror, and then suddenly threw herself furiously between the combatants, wrested the sword from the soldier's hand, broke it in pieces, and threw it away. During the tumult some neighbors came in and separated the men. While in this state of strong excitement, the mother tock up her child from the cradle, where it lay playing, and in the most perfect health, never having had a moment's illness; she gave it the breast, and in so doing, sealed its fate. In a few minutes the infant left off sucking, became restless, panted, and sank dead upon its mother's bosom. The physician, who was instantly called in, found the child lying in the cradle, as if asleep, and with its features undisturbed; but all his resources were fruitless. It was irrevocably gone."

"A lady having several children, of which none had manifested any particular tendency to cerebral disease, and of which the youngest was a healthy infant a few months old, heard of the death of the infant child of a friend residing at a distance, with whom she had been on terms of close intimacy, and whose family had increased cotemporaneously with her own. The circumstance naturally made a strong impression on her mind, and she dwelt upon it the more, perhaps, as she happened at that period to be separated from the rest of her family, and to be much alone with her babe. One morning shortly after having nursed it, she laid it in its cradle, asleep and apparently in perfect health; her attention was shortly attracted to it by a noise, and on going to the cradle, she found her infant in a convulsion, which lasted for a few minutes, and left it dead."

"A mother had lost several children in early infancy from a convulsive disorder. One infant however, survived the usual fatal period; but whilst nursing him one morning, she had been strongly dwelling on the fear of losing him also, although he appeared a very healthy child. In a few minutes after the infant had been transferred into the arms of the nurse, and while she was urging her mistress to take a more cheerful view, directing her attention to his thriving appearance, he was seized with a convulsion-fit, and died almost instantly."

These are interesting cases, and tend to show the great influence the mental affections exert on the secretion of milk, in rendering it deleterious in quality, and unwholesome to to the infant.

Returning then to our subject, you will observe by the analysis, that cow's milk differs from that of woman in the proportions of some of the constituents. that it abounds more in butter, but particularly in cascine, or cheese; and on the other hand, that human milk abounds more in the saccharine principle, or sugar of milk. Now this points out a circumstance from which great benefit may be derived. It is of very frequent occurrence that infants are deprived of the natural nourishment of the mother, and diverse opinions are given relative to the food of infants by persons who really know very little about the matter; one recommends a milk diet, another that the infant must be fed on starch and sugar.

Now to enable the infant to receive a nourishment in every respect similar to the mother, the knowledge of the various proportions which we obtain by chemical analysis, enables us to rectify and produce milk very analogous to human milk from that of the cow, by diluting it with water in the proportion of about half as much again; that is to a pint of milk should be added half a pint of water that has been boiled, which will reduce the cheese principle to the proper proportion; add a small portion of cream to restore the proportion of butter, and then add sugar until the whole is distinctly sweetened, and we have a compound in every respect similar to the milk from the human breast.

To understand the subject of nutrition, allow me to explain to you, that food should, or must embody two great principles; one to nourish, the other to give heat to the body. And, food when consumed, is applied to one or the other of these purposes. Now, in the process of digestion, the constituents of the food are separated, and arranged in three classes.

1st. All that portion derived from animal food, eggs, the curd of milk, the gluten or adhesive portion of wheat and other grain, and whatever in animal or vegetable food can be rendered into *albumen*—of which the best example that can be offered in illustration is the *white of egg*, which is in reality nearly pure albumen—and the principle is therefore called *albuminous*.

2d. All that portion of the food derived from vegetables, starch, sugar, etc., that can be converted into *sugar* in the process of digestion. This principle is, therefore, called *saccharine*.

3d. All the fat, butter, oil, etc., when deprived of the other substances, is left in the state of *oil*, and therefore called *oleaginous*.

Now, of these three the *albuminous* is the *nutrient*, and the *saccharine* and *oleaginous* the *calorifacient*, or heat giving; and chemical analyses show that they vary in composition.

| | ALBUMEN. | | OLEAGINOUS. |
|---|---|---|---|
| | Eggs. | Wheat. | Mutton Fat. |
| Carbon | 55.000 | 55.01 | 78.996 |
| Hydrogen | 7 073 | 7.23 | 11.700 |
| Nitrogen | 15.920 | 15.92 | 9,304 |
| Oxygen, Sulphur, Phosphorus | 22.007 | 21.84 | |

SACCHARINE.

| | Starch Arrow Root. | Sugar from Starch. | Sugar of Milk. | Cane Sugar. |
|---|---|---|---|---|
| Carbon | 44.40 | 37.29 | 40.00 | 42.301 |
| Hydrogen | 6.18 | 6.84 | 6.61 | 6 384 |
| Oxygen | 49.42 | 55.87 | 52.93 | 51.315 |

You will observe that the albuminous or nutrient differs from the saccharine and oleaginous, in containing nitrogen, and sulphur and phosphorus, with carbon, hydrogen and oxygen, while the latter contains only carbon, hydrogen and oxygen—nitrogen being required in those compounds which give strength and formation to the frame.

Now the albuminous, or nutritive, being that portion which affords nourishment to the body, contains those constituents required in the first place for the formation and giving strength to the different portions of the body, and when fully developed, of repairing the general waste continually going on in the system, whether from the usual wear and tear, fractured bones, or the ravages of disease. And the saccharine and oleaginous—the calorifacient or heat-making—to keep up a continual supply of fuel, as it were, that the body may be kept of a regular and proper temperature; for you are no doubt aware that there is a continual supply of carbon, or, in more simple language, of charcoal, required to keep up the natural temperature of the body; and what is not required for immediate use is stored away in the form of fat, to be called into action as occasion requires.

We have seen in the analysis of milk, that that fluid contains butter, cheese, and sugar; consequently we can understand how an infant can thrive so well upon it—the cheese or cascine* of the milk, containing the nitrogenized or nutrient principle, which together with the earths and salts contained in the milk, goes to form the bones, muscles and the different tissues of the body—the sugar, which we have seen by the analysis, contains a large quantity of carbon in its composition, going to keep up the temperature of the infant, while the butter, in the nature of fat, is stored away in a healthy infant, filling up every vacant interstice, causing a roundness and plumpness, the pride and joy of the happy parent.

Now let us mark the difference of the babe that has been denied a milk diet, and is doomed by ignorance to be fed on starch and sugar. You will recollect that these two substances were composed of carbon, hydrogen and oxygen only. By a process of digestion which I need not here enter into, such food is converted into sugar, the carbon of which becomes the fuel by which the temperature of the body is kept up—there being no principle in the food to give albumen, there is nothing taken into the stomach upon which the gastric fluid can expend its solvent powers; the infant is, therefore, much troubled with acid eructations, and the stomach becomes weak and irritable. The want of the nutritive constituent of the food, and the earths and salts, etc., necessary and essential for the formation of the bones and teeth, show a lamentable deficiency in the child's development, and there being no fatty matter to be laid up, the body is emaciated, the countenance is ghastly, the flesh and integuments hang soft and flabby over the bones, no absolute disease can be detected, the child is ravenous and hungry, and the unfortunate babe descends to the tomb a spectre and an object of the most pitiful description. This is no fancy sketch, but one too often met with in the ordinary walks of professional life. And why is it so? Simply because the composition of the human frame, the component parts of our food requisite to produce that frame, and the process of digestion and nutrition, are so little understood.

---

| | Analysis of Caseine from fresh milk. | Albuminous substances found in whey after coagulation with an acid. |
|---|---|---|
| Carbon | 54.825 | 54.96 |
| Hydrogen | 7.153 | 7.15 |
| Nitrogen | 15.628 | 15.89 |
| Oxygen | 22.394 | 21.73 |
| Sulpher | 22.394 | 0.36 |

We now advance from infancy to childhood—and this is a period when the greatest attention is required in supplying nutriment to aid nature in the great work of developing the body. The child is now deprived of the maternal secretion, and dependent on food prepared for its use by the hand of man—perhaps living in a city, and deprived of pure and wholesome milk from the cow. And we know there is a vast disproportion in the quality of milk when the cow is country fed on the natural productions of the farm, and when city fed on slops and grain, the refuse of the brewery.

It is at this age that the great proportion of bony substance is deposited; those of the extremities are lengthened, become more compact and stronger, and the substance of the teeth is deposited in the cells of gelatinous tissue. How necessary is it, then, that this subject should receive the utmost attention of parents. It has hitherto been too much the custom to leave all this, as belonging entirely to nature—as a thing we had nothing to do with. We have been too much in the habit of considering that nature furnished her own materials, and man had nothing to do with her operation. The potter cannot fashion the bowl without the clay, neither can bone be formed without earth. No, my friends, nature must be supplied with the material, which, although offered in the most incongrous forms, she has the power of decomposing, selecting from, and supplying for the various purposes required; one portion, as we have already stated, to act as fuel in keeping up the temperature; another portion she selects to add to the flesh, the muscle, skin, and different tissues; and the earths which are held in solution, she carries away by vessels adapted for that purpose, and deposits them atom by atom, until they are so compressed, so strongly compacted together, as to become what we call *solid bone*; and all this so wonderfully wrought, that as we have seen, small tubes are left in the hard stony formations both of the bones and of the teeth, that nourishment may be supplied them, holding in solution the material of which they are composed, that the natural waste and decay may be replaced, and injuries repaired.

It is to this nutrition, and the earthy matter of which the bones and teeth are composed, a deficiency of which is attended with results so deplorable, that I particularly wish to call your attention.

To what can we attribute the calamity which too often befalls the young? I allude to distorted spines, where the bones composing the spine, instead of forming a column, allowing the body to be erect and dignified, are zigzag in their course, causing one shoulder to bulge out, and the opposite side to bend or double upon itself. This deformity has been long understood to arise from a deficiency of *lime* in the composition of the bones of the vertebræ, allowing them to fall, press upon, and injure each other, destroying the beauty of the fabric, and the health and comfort of the individual.

Now let us take a glance at the inhabitants of two countries, natives of which are no strangers on this continent. I take them as examples, because the food of the *common people* of those countries, is well known to be of the most common kind. I allude to natives of Scotland and Ireland —the principal food of one being *oatmeal*, and of the other, *potatoes*. We have heard a great deal of the famishing poor of those countries, and particularly of the latter — of the misery and wretchedness seen in every hovel; and there cannot be a doubt that famine walked through the land, when the blight and rot despoiled them of their potatoe crop, on which, for so long a period, they depended as the great article of food. Now, allowing all this—allowing, in the *best seasons* the chief article of subsistence has been potatoes for breakfast, dinner and supper; glad indeed many of them to get a little animal food once a week to dinner, or even far more seldom—I now ask, what number, in the thousands of emigrants from that country who yearly arrive at our ports, are there that show a constitution weak, fragile, and wanting in physical strength? Many, no doubt, arrive, worn down by disease and suffering, and in the last stage of debility; but let them recover from that state, and the robust frame and healthy constitution will be again developed; the bones are strong, the teeth undecayed, and the muscular energy only wanting opportunity to display itself; — in fact, when we wish to denote strength in woman, we use the familiar phrase, "strong as an Irish woman;" and all this from being reared on *potatoes*. But then, if we examine the analysis of the potatoes, we shall find contained in 100 parts of dry potatoes,—

| | |
|---|---|
| Carbon | 41.1 |
| Hydrogen | 5.8 |
| Nitrogen, Oxygen | 45.1 |
| Ashes | 5.0 |

Here we see that potatoes not only contain the nutriment but the earthy constituents.*

NUTRITIVE ELEEENTS.

| | | | | | |
|---|---|---|---|---|---|
| 100 lbs. | Wheat Bread | contains | 30 lbs. | | |
| " | Flesh | " | 21 lbs. | | |
| " | Fresh Beans | " | 80 lbs. | casein and starch. |
| " | Peas | " | 83 lbs. | |
| " | Lentils | " | 94 lbs. | |
| " | Potatoes | " | 25 lbs. | albumen, starch and sugar |
| " | Carrots | " | 14 lbs. | albumen with sugar. |
| " | Beets | " | 8 lbs. | |

But we have a stronger and more healthy race yet, from Scotland and the north of Ireland, who are generally descendants of the Scotch, and continue, in a great measure, the same means in rearing the young. Now, *a* principal, I will not say *the* principal food of the youth of Scotland, high and low, rich and poor, except in the larger cities, amongst those who class themselves as more refined and more civilized, but who number few in proportion, consists. for breakfast, at least, of oatmeal—that is, porridge and milk ; and milk, potatoes, and wheaten, oaten, and peas bread, or *bannocks*, at other times of the day. Animal food amongst the poor is a rarity ; a meat dinner on Sunday *only*, being common. Even, among the youth of the better class, butchers meat, or animal food, is by no means a principle article of subsistence. And I would particularly remark that *Scotch oatmeal* (the oatmeal generally used throughout Scotland) is course, and contains much of the bran which invests the oat — containing, as it does, a large proportion of the earthy constituents required for the production of bone. Analysis of 100 parts of dried oats gives:

| | |
|---|---|
| Carbon | 50 7 |
| Hydrogen | 6.4 |
| Oxygen | 36.7 |
| Nitrogen | 2.2 |
| Ashes | 4.6 |

I may here casually remark, that the advantage to be derived from this wholesome food has not escaped the observation of her majesty, Queen VICTORIA, who appears in the

* According to a memorial presented to the French minister, on the proportions of nutriment of the means of living, by Dr. GLASER, we find potatoes taking no mean rank.

multiplicity of her public duties, not to lose sight of the equally sacred duties of a mother—and we hear of her son, the heir to the crown of Great Britain, being as fond of his oatmeal porridge as the meanest peasant child in Scotland.

I rather doubt, if parents generally have given to this subject the attention to which it is entitled. I trust, however, that those who have followed me thus far, may be impressed with its importance. We cannot shut our eyes to the complaint which so generally prevails, of decayed teeth—and a moment's reflection will call to mind the number of the young and beautiful who are prematurely hurried to the tomb, ere yet the bud has expanded into the full-developed flower. Nay, comparing the two countries, the statistics, of life and death communicate to us also the important fact, that while the greatest mortality shows itself in England in infancy and childhood, on this side of the Atlantic, it is found at a more mature age.

Neither has the tendency of the physical organization of woman on this continent to degenerate, escaped the observation of one of our greatest medical philosophers in this country,* who regards this retrogression as a national calamity, and impresses upon his students the importance of the subject, and the propriety of their attention in attempting to arrest it; and he particularly specifies the great object to be gained in the use of bran-bread, made from unbolted flour. On this head, I shall have more to say hereafter. With these observations, let us now direct our attention to what can be offered in remedy of this evil.

We have already stated, that in no country in the world are children more beautiful or more lovely—healthy in complexion, quick, smart, and intelligent—active, sprightly, and playful in their dispositions. Now, in the period from infancy until the child becomes mature—let us, at all events, say until thirteen or fourteen years, and even to a more advanced age—there is a continued growth—a continual deposition of organic and inorganic or earthy particles, which are required for the formation of bone, teeth, flesh and every part of the human body. I have shown you that the essential ingredients for these several formations are all found in the milk of the mother; consequently, as long as the infant is deriving nourishment from the mother, she ought to par-

* Dr. Jackson, of Philadelphia.

take of good, wholesome, nourishing food — that the blood deriving these principles from the food, may be able to supply them in turn to the milk from which it is secreted. So long then, as, the child is thus nourished, so long is it safe, take of good, wholesome, nourishing food — that the blood, and the rudiments or foundation of a robust frame is laid.—And if we are to expect, in future life, the stalwart frame of man, or the enduring, firmly knit, compact, and healthy physical constitution in woman, the organic and inorganic or earthy compounds of which that frame is composed must not be denied—Nature must be supplied, or Nature will fail.

It is not for me to dictate to any parent what should be the food of his child— it is enough that I point out for their information, what may be required to give, what in common language is called "bone and sinew," to their offspring. It is necessary then that the food of children shall contain :

1st. Aliment, having the *calorifacient* or heat sustaining principle. And this is contained in quite sufficient quantity in the usual food—in milk, wheaten bread, potatoes, arrow-root, Indian corn, (as mush, hominy, or corn-bread,) in most vegetable matter, and in sugar.

2d. Aliment containing the *nutrient* principle. And this is contrived in animal food—the lean of beast, bird, and fish —in milk, eggs, wheat, rye, potatoes, beans, etc , etc.

And, 3d. Aliment containing the inorganic or earthy constituents—on which depends strength of frame, and from which are formed the bones and teeth of the individual. And these are contained in milk, eggs, animal food, and particularly in wheat, rye, oats, potatoes, etc.*

* On this subject, I extract the following from CARPENTER's Phisiology, p. 488 : "These substances are contained, more or less abundantly, in most articles generally used as food ; and where they are deficient, the animal suffers in consequence, if they are not supplied in any other way. Thus, common *salt* exists, in no inconsiderable quantity, in the flesh and fluids of animals, in milk, and in eggs ; it is not so abundant, however, in plants, and the deficiency is usually supplied to herbivorous animals by some other means. *Phosphorus* exists also in the yolk and white of the egg, and in milk—and it abounds, not only in many animal substances used as food, but also (in the state of phosphate of lime or bone earth) in the seeds of many plants, especially the *grasses*. In smaller quantities, it is found in the ashes of almost every plant. *Sulphur* is derived alike from vegetable and animal substances. It exists in flesh, eggs, and milk ; also in the azotized compounds of plants ; and (in the form of sulphate of lime) in most of the river and spring-water that we drink. *Iron* is found in yolk of egg, and in milk, as well as in animal flesh ; it also exists in small quantities in most vegetable substances used as food by man—such as potatoes, cabbage, peas, cucumbers, mus-

Of the inorganic constituents contained in wheat, (and the same may be said of the other *cereal* grains,) I have already alluded to the benefit to be derived from using bread made of unbolted flour. On this subject, allow me to refer to the difference of flour having much of the bran remaining, and superfine flour, or that in general use throughout this country, and on which Prof. Johnson has made the following curious but practical observations. Examining wheat and flour, as to the amount of the nutrient or muscular matter, the fat-forming principle, and the bone and saline material, contained in grain in different states, he found that

| | Muscular Mat. | Fat Prin. | Bone & Sal |
|---|---|---|---|
| In 1000 lbs of whole grain.... | 156 lbs. | 25 lbs. | 170 lbs. |
| " " fine flour...... | 130 lbs. | 20 lbs. | 60 lbs. |
| " " bran.......... | ....... | 60 lbs. | 700 lbs. |

Taking the three substances together, according to Prof. Johnson, of a thousand pounds, the three substances contain, of the ingredients mentioned,—

| | Whole Grain. | Fine Flour. |
|---|---|---|
| Of muscular matter.......... | 156 lbs. | 130 lbs. |
| Of bone material ............ | 170 lbs. | 60 lbs. |
| Of fat..................... | 28 lbs. | 20 lbs. |
| | 354 lbs. | 210 lbs. |

Accordingly, the whole grain is one-half more nutritious than fine flour.* It also shows the very great proportion of *bone material*, that is, *earthy constituents*,—contained in the bran: no less than 700, out of a thousand parts, or a *little more than two-thirds* of the whole. Now, by reference to the same work, we find, in a communication from a Mr. Bentz, the difference in weight of a barrel of flour, without the bran, and when only the outer coating of the wheat is taken off. He says, " The weight of the bran or outer coating would, therefore, in the common superfine flour, constitute the *offal*, tard, etc. *Lime* is one of the most universally diffused of all mineral bodies; for there are few animal or vegetable substances in which it does not exist. It is most commonly taken in, among the higher animals, combined with phosphoric acid: in this state it exists largely in the seeds of most grasses, and especially in wheat flour. If it were not for their deficiency of *lime*, some of the leguminous seeds (peas) would be more nutritious than wheaten flour; the proportion of azotized matter they contain being greater. A considerable quantity of lime exists, in the state of carbonate and sulphate, in all hard water."

* Patent Office Report, 1847, p. 116.

weighing only 5¼ lbs. to the barrel of flour, whilst the ordinary weight of offal is from 65 to 70 lbs. to each barrel of flour, showing a gain of from 59¾ to 65 lbs. of wheat in every barrel of flour." Now, if we estimate the earthy constituents to be two-thirds of the offal or bran, we must consider that there is an actual loss of these important constituents, which might be reserved, in every barrel of flour, of 40 lbs.

*Again*, if we estimate, (according to the average of the consumption of flour to the amount of population, as one barrel to each individual,) that every child shall consume annually only half a barrel of flour, then we find, that by the use of the superfine flour, as commonly used in families, the child is deprived yearly of twenty lbs. of those earthy substances which are required to form the bones and the teeth. When we speak of a child consuming half a barrel of flour annually, it appears a large quantity; but when we reduce the same to a daily allowance, we find that it is little more than 4 oz. or 4⅓ oz.; and every parent must know that this would be a very small amount to limit children. Yet we see how large a quantity of the bony material would be added, if unbolted flour was used instead of the present superfine flour. I may here add, that the oatmeal used in Scotland, already referred to, contains the bran or inorganic constituents, while the oatmeal used in England is deprived of it. Now this is a great loss of the most valuable constituents in only one of the principal articles of the food of children; and if we allude to another article, which is largely used on this continent,— I mean Indian corn, — (and I may also add the fat of meat, both of which, children, if allowed, will partake of very freely,) we shall find that both of these abound more in the calorifacient, or heat-sustaining principle, and for the deposition of fat, than the nutrient; and that they are quite deficient of the earthy material, *of lime*—that material on which so much depends the proper structure of the teeth. Analysis of Indian corn shows the following composition—as taken from Mr. Salisbury's prize essay—read at the New York Agricultural Society, for 1849:

Whole kernel.

| | |
|---|---|
| Starch | 50.64 |
| Sugar and Extractive | 7.46 |
| Sugar | 1.50 |
| Fibre | 6.28 |
| Matter separated from Fibre | 0.05 |

| | |
|---|---|
| Albumen | 8.64 |
| Caseine | 1.70 |
| Gluten | 4.56 |
| Oil | 4.00 |
| Dextrine or Gum | 4.84 |
| Water | 10.22 |
| | 99.89 |

Ash of the kernel constituting about two per cent.

| | |
|---|---|
| Carbonic acid | a trace. |
| Silicic " | 1.450 |
| Sulphuric " | 0.206 |
| Phosphoric acid | 50.955 |
| Phosphate of Iron | 4.355 |
| Lime | 0.150 |
| Magnesia | 16.530 |
| Potash | 8.286 |
| Soda | 10.908 |
| Chloride of Soda | 0.249 |
| Organic acid | 3.400 |
| | 97.000 |

This is a more elaborate analysis — far more minute than any analysis we have had of any of the articles of food—in fact, more minute than satisfactory; for the analysis of the whole kernel does not exhibit any amount of inorganic constituent; and when the whole was converted into ashes, we find that the *lime* only amounts to *the one sixth of one part* in a hundred. Now, on inquiry, I find, on the authority of a very intelligent miller of this city, that in grinding corn, the bran, or thin skin of the grain, is detained in forming it into corn-meal; consequently it is deprived of even that portion more particularly containing the earthy constituents. This gentleman in conversation mentioned an important fact, relative to this deficiency of lime in corn. To the best of my recollection, he observed, "This stands to reason; for ten years ago, all the lower part of Jersey grew excellent corn, but would not grow wheat; but since the introduction of *lime* as a manure, they have raised considerable wheat crops." Now the fact is, it is not the habit or food of this plant, even had *lime* been in the earth; and magnesia and the saline manures are recommended to the agriculturist as best suited for its proper development.

It is generally looked upon as invidious, and one is more likely to incur odium, than to receive credit for saying one word against a food which stands so high in public estimation, and is so universally used over this continent. Yet it must not, for one moment, be supposed that I condemn the use of Indian corn, in its various forms of mush, hominy, bread, or pudding, as an article of diet; far from it. But containing, as it does, a large proportion of starch and fatty matter, rather a small proportion of the nutrient principle, and quite a deficiency of the inorganic or earthy constituents, I consider it as valuable, as a light diet, for heat-sustaining purposes only, and therefore a desirable adjunct *to other food*, containing more nutriment and a due proportion of the earthy constituents.

As an example or illustration of the want of the nutrient principle in corn or corn-meal, I may here allude to the effects I have seen in the West Indies; where, in a dearth of the ordinary provisions on which prisoners were fed, corn-meal was substituted; corn-meal and salted herrings, fish, etc., constituted their food. Now the effect was, that all the prisoners lost their natural strength; at the same time, they became fat and bloated, inclining to dropsy: and this was not the effect of incarceration; for the prisoners were engaged in road-making, trimming fences, etc.; consequently, in a healthy and exhilarating employment.

In reference to our domesticated animals, it may be asked, Why is corn so useful, as an article of food, to animals generally—horses, hogs, sheep, etc.? I have already shown that the overplus of the calorifacient food, after what may be required for sustaining the temperature, is stored away in the form of fat. Now, if we instance the horse; corn is generally, if not always, given as an adjunct to his more usual food, hay. And we find by analysis, that grass or hay contains not only the nutrient principle, but the inorganic constituents required in the formation of bone, etc.

One hundred parts of dry hay contain—

| | |
|---|---|
| Carbon | 45.8 |
| Hydrogen | 5.0 |
| Oxygen | 38.7 |
| Nitrogen* | 1.5 |
| Ashes,† | 9.0 |
| | 100 |

Thus, the hay gives to the animal strength in bone and muscle, while the corn supplies additional heat-sustaining

* Fifteen pounds of such hay, containing oz. 3.095 of nitrogen.
† These ashes having a good proportion of lime.

properties, and lays by, in the form of fat, the overplus as a reserve. The harder the horse is worked, the more corn he can bear; the great proportion of the carbon being carried off by the lungs, and the hydrogen and oxygen, as water, in exhalation and perspiration. But if the same quantity is given to a horse at rest, it overloads him with fat, which, in his case, accumulates more internally, or around the internal organs, and will, in course of time, induce disease; while in the pig, under similar circumstances, the fat is laid on externally, if I may so speak, giving the rich fat pork of our markets. And here I would again remark, that no farmer would consider it necessary or essential to give corn to a young colt or horse, until required to work; nay, so careful is nature, in appropriating just so much and no more of any constituent that may be required, that the food of the young horse should be more nutritious than heat-sustaining; and that there shall be no superfluity to store away fat, we find by analysis, that the milk of the mare has little or no butter, in fact only traces of it, in its composition.‡ What a lesson in the animal economy is here given, and what a practical illustration of the requirements of the young of that and other animals!

Again, it may be contended, that among the beautiful children we see on every hand, there is no want of those who are fat and hearty. It is not *fat* we want—it is bone and muscle—with so much fat only as shall give firmness to the flesh and plumpness to the figure. Fat, although it enters intimately into union with the other component parts of bone and muscle, cannot be transformed either into the inorganic constituents of bone or teeth, or into muscular fibre; these must be contained in the food consumed, in the first place, and thence transfered to the blood.

How necessary, then—how important it is — if we expect to give strength and vigor to the constitution, that the food, in the first years of infancy and childhood, when the formative process is going on, should receive some further attention than has hitherto been given to it; and if our youth—

‡ ANALYSIS OF MARE'S MILK.

| | |
|---|---|
| Water | 896.3 |
| Butter | Traces. |
| Caseine | 16.2 |
| Sugar of Milk, Extractive Matters, and Fixed Salts | 87.5 |
| | 1000. |

if our young females have hitherto been deprived of the necessary constituents for the full development of every portion of the body—can we wonder that a woman should be the delicate and fragile being she is, or that by the decay which assails the teeth in early life, she should be deprived of an ornament of so much value ? If this state of things can be altered—if the physical constitution of woman in America can be saved from further degeneracy — a purpose may be effected, of consequence even in a national point of view ; for it is to the healthy and vigorous constitution of woman that we must look for a race of hardy, vigorous and enterprising freemen.

In conclusion, I would briefly state, that this is a matter in which professional aid can avail little ; it lies at the door, and must be the work of parents generally. It is for them to understand the great value to be attached to the food on which their children subsist—that it shall be wholesome and nutritious, and abounding in the earthy compounds so absolutely necessary to their proper development. If the chief articles of food have hitherto consisted of compounds made of superfine flour, corn meal, and the fat of meat, let there be substituted in their stead, bran-bread, milk, eggs, the lean of meat, and potatoes ; let more attention be given to the nutrient quality of the food ;—let there be no deficiency of those articles containing the earthy material, that the bones and teeth shall not be deficient in those constituents so necessary in their composition and structure; and I should be inclined to hope that the evils which now exist will be lessened, and the physical organization of succeeding generations be equal to that of any nation upon earth.—*Med. & Surg. Rep.*

